# Brain health strategies in middle-aged individuals with mild neurocognitive disorder: a scoping review

**DOI:** 10.1016/j.tjpad.2026.100657

**Published:** 2026-08-13

**Authors:** Joshi Dookhy, Yvonne McCague, Jim Hickson, Diarmuid Stokes, Thilo Kroll, Kate Frazer

**Affiliations:** a School of Nursing, Midwifery and Health Systems, University College Dublin, Dublin, Ireland; b Neurology Department, University Hospital Galway, Galway, Ireland; c Regional Hospital Mullingar Co., Westmeath, Ireland; d Children’s Health Ireland, Temple Street, Dublin, Ireland; e James Joyce Library, University College Dublin, Dublin, Ireland

**Keywords:** Mild cognitive impairment, Mild neurocognitive disorder, Brain health, Equity, Middle-aged, Dementia risk reduction

## Abstract

Brain health refers to optimal brain functioning and integrity, shaped by complex cognitive, psychological, and social determinants. Individuals with mild neurocognitive disorder (mNCD) are at higher risk of developing dementia. Prioritising brain health is essential throughout the lifespan, particularly at midlife when health risks often emerge alongside heavy life responsibilities. International frameworks emphasise addressing modifiable risk factors across the lifespan to reduce dementia risk, yet little is known about the strategies middle-aged individuals with mNCD use to maintain brain health in everyday life. Understanding how people in midlife interpret and act on information about brain health is essential to align clinical care and prevention policies with lived experience. This scoping review followed the Joanna Briggs Institute methodology and mapped data published from August 2015 to 2025 using the 2024 Lancet Commission on dementia and the Cochrane PROGRESS-Plus equity framework. The primary aim of this review was to map the range and characteristics of non-pharmacological brain health strategies used by middle-aged individuals with mNCD to reduce the risks of developing dementia. After screening 6349 records, 17 articles were included. The results are presented in three groups: brain health strategies, facilitators and barriers across strategies, and the equity analysis. Strategies were classified as formalised interventions and self-directed practices. Facilitators and barriers varied according to population characteristics and the strategies used. Equity gaps included limited representation of study participants from rural and low-socioeconomic settings, reduced generalisability to male populations, and underrepresentation of minoritised ethnic groups. Clinicians, service planners, and policymakers should prioritise equity-oriented brain health strategies co-designed with middle-aged individuals. Equity and co-design should be explicitly integrated within global brain health policies and agendas.

## Highlights

• Midlife mNCD provides a critical window for brain health risk modification.
• Formalised interventions and self-directed strategies report benefits to cognitive function and psychological well-being.
• Engagement with these strategies is influenced by a complex interplay of psychological, cognitive, and logistical factors, as well as social determinants of health.
• Equity gaps limit real-world brain health reach, engagement, and scalability.

## 1 Background

Mild neurocognitive disorder (mNCD) is characterised by a measurable decline in one or more cognitive domains that represents a change from prior functioning but does not significantly interfere with an individual’s daily activities [1,2]. The terms ‘mNCD’ and ‘mild cognitive impairment’ (MCI) have often been used interchangeably [3,4]. However, mNCD encompasses a wider range of cognitive impairment aetiologies, making it more applicable to diverse populations [2]. This condition reflects a prodromal stage of dementia, although trajectories are heterogeneous, and some cases remain stable or revert to prior cognitive functioning [5,6].

Over the past decade, dementia prevention has been increasingly prioritised as a rapidly growing global public health challenge [7,8]. Concurrently, epidemiological works on dementia prevention estimated that approximately 40–45% of dementia cases can be reduced or delayed by addressing a set of modifiable risk factors [9]. These include behavioural (physical inactivity, smoking, excessive alcohol consumption), cardiometabolic (obesity, hypertension, diabetes, elevated LDL cholesterol), psychosocial (depression, social isolation, low educational attainment), head injury, sensory (hearing impairment, untreated vision loss), and environmental exposure (air pollution). The term ‘brain health’ is generally preferred over ‘dementia risk-reduction,’ which is often seen as stigmatising. ‘Brain health’ resonates with individuals across different age groups and fosters proactive engagement in long-term strategies, behavioural changes, and offers a more acceptable and empowering way to discuss prognosis and address risk factors [[10], [11], [12], [13]].

Brain health is defined as a state of brain “functioning across cognitive, sensory, social-emotional, behavioural and motor domains, enabling a person to realise their full potential over the life course, irrespective of the presence or absence of disorders” [14]. Pathological processes underlying late-life dementia typically begin decades before clinical diagnosis, with accumulating evidence showing that midlife (typically 40–65 years) is a critical phase for modifying risk factors [15,16]. Most modifiable risk factors, such as mood changes, poor sleep, and hearing loss, emerge and are most amenable to change at midlife [17,18]. Vascular, metabolic and mental health also cluster during these years, alongside work and caregiving responsibilities that can both support and constrain health behaviours [19,20]. Brain health, however, is not merely a biological or pathological matter; it is fundamentally shaped by a complex network of real-world socio-economic and environmental factors [7]. Social determinants of health (SDoH) are the social, economic, and environmental conditions that shape health risks and access to care [21], thereby influencing who can engage with and benefit from strategies to optimise brain health. SDoH can be characterised using the PROGRESS-Plus framework to synthesise equity-relevant characteristics (Place of residence, Race/ethnicity/culture/language, Occupation, Gender/sex, Religion, Education, Socioeconomic status, Social capital, and “Plus” factors such as age and disability) [22].

Pharmacological approaches to dementia prevention typically target pathology or symptoms with cholinesterase inhibitors, insulin, and emerging monoclonal antibodies [23,24]. These have shown minimal to negligible effects on MCI progression or have limited eligibility for patients [[25], [26], [27]]. Non-pharmacological strategies are therefore regarded as the cornerstone of current brain health initiatives. However, these approaches mainly focus on older adults or the general healthy population [28,29]. Past research has primarily investigated multimodal interventions delivered in trial settings and in high-income countries [30,31]. This scoping review, therefore, sought to understand how middle-aged individuals with mNCD, who face a higher risk of dementia, manage daily responsibilities while maintaining brain health in real-world environments. The main aim was to map the range and characteristics of non-pharmacological brain health strategies used by this group. The secondary aims were to map and analyse how these individuals select and engage with these strategies, considering barriers, facilitators, and equity-related issues.

## 2 Methods

This scoping review followed the Joanna Briggs Institute (JBI) methodology and drew on the framework proposed by Arksey and O’Malley [32], as refined by Levac and colleagues [33]. Reporting adheres to the Preferred Reporting Items for Systematic Reviews and Meta-Analyses extension for Scoping Reviews (PRISMA-ScR) checklist [34]. Ethical approval was not required.

### 2.1 Protocol and registration

The protocol was developed a priori and registered on HRB Open Research (https://hrbopenresearch.org/articles/8-63/v3). The data-charting form was refined iteratively during pilot extraction, as anticipated in the protocol, to capture additional descriptive variables that emerged as relevant. These refinements did not change the eligibility criteria, population definition, study-selection process, or review objectives. No substantive deviations from the protocol were made.

### 2.2 Search strategy

The search strategy was initially developed for PubMed via MEDLINE by a subject librarian (DS) working together with a member of the review team (JD). A limited search was conducted in July 2025 to identify relevant articles. Text words in the titles, abstracts and index terms used to describe these articles were analysed to refine the search strategy. Once finalised, the search strategy was adapted for Embase via Elsevier, CINAHL via EBSCOhost and PsycINFO. Search strategies combined controlled vocabulary (e.g., MeSH) with free-text terms synonymous with mild neurocognitive disorder, brain health, midlife, and non-pharmacological strategies. All searches were conducted on 14th August 2025.

The search was limited to studies published in English from January 2015 onwards to ensure the evidence reflects contemporary definitions, methodologies and technological advances in brain health research, since the earliest record pertaining to ‘brain health’ being discussed and recognised as a concept stems from the 2015 International Association of Gerontology and Geriatrics and its Global Aging Research Network consensus panel [35]. The language limiter was applied to ensure accurate screening of abstracts based on the review team's language capabilities and translation constraints, and to ensure consistent data extraction and synthesis. We also examined the reference lists of all included articles to identify additional studies. Grey literature searches targeted guidelines, reports, and evaluations from international organisations and brain health strategies. The full search strategy is provided in Supplementary Table 1.

### 2.3 Eligibility criteria

The Population-Concept-Context (PCC) framework [36] was used to define the eligibility criteria and to ensure alignment with our exploratory research aims within a specific population and context. The population of focus was middle-aged adults with mNCD, with midlife operationally defined as being aged 40–65 years [37]. Studies frequently recruited participants across broader age ranges. Therefore, eligibility was determined by their recruitment criteria and reported participant age characteristics, including adults aged 40–65 years and studies with mixed-age samples overlapping with the midlife period. The reported age characteristics, including the mean age, standard deviation or age range, were used to describe the extent to which each sample represented the midlife population. mNCD was treated as an aetiologically inclusive syndrome, allowing the review to capture relevant studies across different clinical populations. We excluded studies focusing exclusively on participants with younger-onset or established major neurocognitive disorders. The central concept was non-pharmacological approaches, including lifestyle modifications, cognitive and social activities, psychological and coping strategies, digital tools, and engagement with services. We focused on real-world settings, including community, occupational, or clinical environments in any country. Controlled laboratory paradigms without clear translation to everyday practice were excluded as they may not reflect the practical experiences of adopting or maintaining brain health.

### 2.4 Data screening

Search results from each database were imported into EndNote 21 and then uploaded to Covidence (www.covidence.org) [38], as detailed in Supplementary Table 2. Each title and abstract screening was conducted independently by at least two reviewers (JD, YMcC, KF, and JH) against predefined eligibility criteria. Articles deemed potentially relevant by reviewers progressed to full-text review, which was completed independently by three reviewers (JD, JH, and YMcC). Disagreements at each stage were resolved through discussion and, when necessary, mediated more conservatively by another reviewer (TK). Twelve protocols that progressed to full-text screening were separately searched to determine whether the completed studies could be included. Unfortunately, six could not be found, and the others no longer met the age criteria for this scoping review.

### 2.5 Data extraction and synthesis

Data were extracted by three reviewers independently using a data extraction template in Microsoft Excel. JH and YMcC extracted 24% of the included studies, and JD completed the rest. Extracted information included study characteristics (e.g., sample size, study design), participant characteristics (e.g., age, diagnostic criteria of MCI), bibliographic details (e.g., author, year), description of strategies (e.g., type, duration), modifiable factors of brain health addressed, PROGRESS-Plus equity factors, and key barriers or facilitators of engaging in interventions. We completed a descriptive and narrative synthesis approach consistent with JBI guidance for scoping reviews.

## 3 Results

### 3.1 Search results

**Fig. 1:**
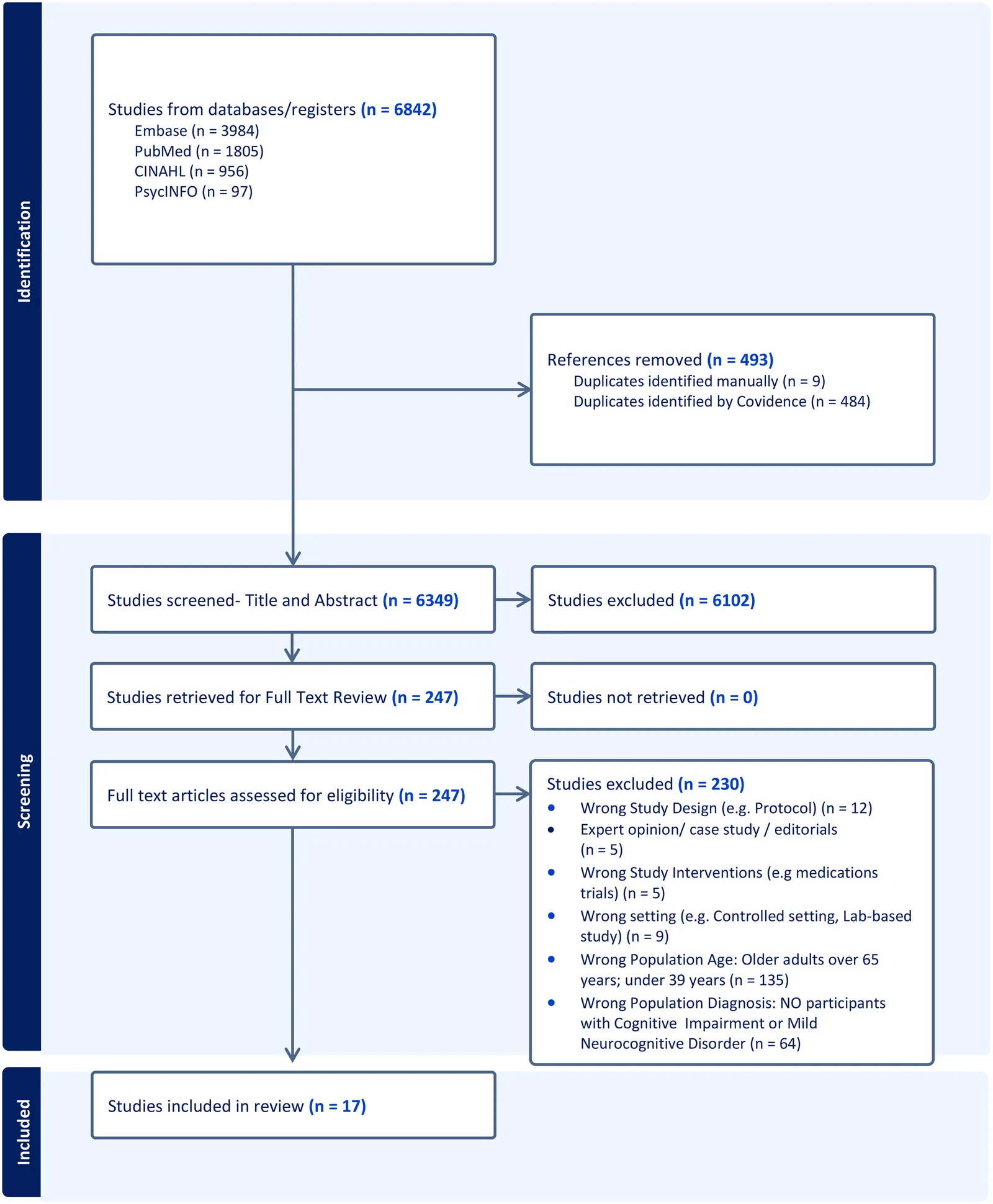
PRISMA-ScR flow Diagram.

The study selection process and search results are presented in a PRISMA-ScR flow diagram (Fig. 1). A total of 6842 articles were retrieved from the initial search and uploaded to Covidence; after deduplication, 6349 unique studies were included in title and abstract screening. Of these, 247 articles were retained for full-text screening, and 17 studies are included in this review.

### 3.2 Study characteristics

Table 1 summarises the characteristics of the included studies. Predominant study designs include randomised controlled trials (RCTs) (n = 7, 41.2%), quasi-experimental studies (n = 3, 17.6%), and observational studies (n = 7, 41.2%). Geographical location of studies includes Asia (n = 7, 41.2%) [[39], [40], [41], [42], [43], [44], [45]], Europe (n = 6, 35.3%) [[46], [47], [48], [49], [50], [51]], North America (n = 3, 17.6%) [[52], [53], [54]] and Africa (n = 1, 5.9%) [55]. The total number of study participants across the 17 completed studies was 14,486. The reported settings included community (n = 6, 33.3%), hospital clinics (n = 6, 33.3%), rehabilitation centres (n = 4, 22.2%), and one study was located in a workplace [45]. Most studies (n = 15, 88.2%) included mixed-sex participants, and two included only females [39,48]. A gender imbalance was observed across the literature, with ten studies reporting a predominantly female participant base.

**Table 1:** Characteristics of the 17 studies that met scoping review criteria, organised alphabetically.

Authors, Year of publication	Country	Study design	Research Setting	Population	Sample size	Study’s recruitment age criteria^1^	Actual age reported (Mean± SD, where available)^2^	Primary Objective
An & Wang, 2023	China	Longitudinal observation study	Community-based	Individuals enrolled in the CHNS	n = 4734	≥55 years	63.9 years	To assess the longitudinal relationship between whole egg consumption and cognitive function in older Chinese adults.
Baetge et al., 2023	Germany	RCT	Hospital outpatient clinic	Individuals with MS	n = 50	≥18 years	MaTiMS: 51.85±6.60 years MaTiMS modified: 55.17±6.61 years	To compare the effects of a standard metacognitive training program versus a modified version on neuropsychiatric and cognitive outcomes in individuals with multiple sclerosis.
Bampa et al., 2024	Greece	Cross-sectional observation study	Online setting	Individuals with existing amnestic MCI & healthy controls	n = 100	>50 years	62.76 years	To examine how individuals with amnestic mild cognitive impairment perceive their ability to handle daily cognitively challenging situations and their use of compensatory cognitive strategies.
Chen et al., 2025	China	Cross-sectional observation study	Hospital neurology clinics	Individuals with MCI due to Alzheimer’s disease	n = 604 patient/ caregiver dyads	≥50 years	67.67±8.18 years^3^	To investigate the level of dementia risk-reduction lifestyle in patients with mild cognitive impairment and identify how patient and caregiver factors influence it.
Currenti et al., 2021	Italy	Cross-sectional observation study	Community-based	Individuals enrolled in the MEAL study	n = 883	>50 years	Multiple range for study population:61.5–65 years	To investigate the association between daily time-restricted feeding and cognitive status in a cross-sectional cohort of older Italian adults.
Fitriana et al., 2020	Indonesia	Quasi-experimental, pre-post with control	Research university setting	Community-dwelling individuals	n = 32	44–61 years	Aerobic: 51.88±4.17 years Control: 51.75±4.95 years	To determine the effect of a 16-week aerobic exercise regimen on cognition, physical fitness, and Apolipoprotein E levels in women with mild cognitive impairment.
Li et al., 2018	China	Quasi-experimental, with multi-year follow-up	Workplace	Individuals with existing hypertension	n = 510	None	45.6 ± 13.4 years	To analyse the impact of a 2-year lifestyle intervention on mild cognitive impairment in an occupational cohort in China.
Li et al., 2024	China	Cross-sectional observation study	Community-based	Individuals enrolled in The Lifestyle and Healthy Aging of Chinese Square Dancer Study	n = 2621	≥45–55 years	63.71 years	To evaluate associations between aquatic food consumption, long-chain n-3 PUFA intake, and blood mercury levels with cognitive performance in middle-aged and older adults.
Lu et al., 2025	China	Cross-sectional observation study	Community-based	Individuals enrolled in The Lifestyle and Healthy Aging of Chinese Square Dancer Study	n = 4335	≥55 years	63.61 years	To assess the relationship between adherence to a Chinese-adapted MIND diet and cognitive function (global and specific domains), as well as the prevalence of MCI, in middle-aged and older Chinese adults.
Maggio et al., 2022	Italy	RCT	Rehabilitation hospital	Individuals with MS	n = 60	19–74 years	50±11.4 years	To evaluate the effect of semi-immersive virtual reality training on neuropsychological and motor recovery in patients with multiple sclerosis, compared to conventional cognitive training.
Melis et al., 2022	Belgium	RCT	Mindfulness centre (part of university)	Breast cancer survivors (post-chemotherapy)	n = 117	18 to 65 years	48.4 ± 8.2 years	To test whether a mindfulness-based intervention can reduce cancer-related cognitive impairment in breast cancer survivors with cognitive complaints, in comparison to a physical exercise program and a control.
Moreu-Valls et al., 2025	Spain	RCT	Hospital neurology clinics	Individuals with early-to-middle stage HD	n = 44	None	Multiple range for study population: 50.07±11.11 - 58.31±6 years	To investigate the effects of computerised cognitive training and music therapy on cognition, functional status, and brain structure in early-to-middle-stage Huntington’s disease.
Nweke et al., 2022	Nigeria	RCT	Hospital HIV clinic	Individuals with HIV	n = 73	<65 years	Intervention:45.8 years Control: 46.2 years	To determine the effect of a 12-week moderate-intensity aerobic exercise program on cognition in people with HIV-associated neurocognitive disorder.
O' Neil-Perozzi & Hsu, 2016	USA	Quasi-experimental with intervention & control arms	Community-based supported living facilities	Individuals with existing MCI due to acquired brain injury	n = 14	26–62 years	Experiment: 51.3 ± 11.5 years Control: 46.9 ± 10 years	To explore the feasibility and effects of participation in a computerised cognitive fitness exercise program by adults with chronic cognitive impairments post acquired brain injury
Parial et al., 2023	Philippines	RCT	Community centre	Individuals with existing MCI	n = 60	≥55 years	63.8 ± 5.24 years	To evaluate the preliminary efficacy of a dual-task Zumba exercise intervention on cognitive outcomes in people with mild cognitive impairment.
Pettigrew et al., 2019	USA	Longitudinal observation study	Hospital	Individuals enrolled in the BIOCARD study who progressed to MCI	n = 189	None	56.6 ± 8.5 years	To examine whether current engagement in physical, cognitive, and social activities is related to prior cognitive trajectories.
Vas et al., 2016	USA	RCT	Community-based	Individuals with existing MCI due to traumatic brain injury	n = 60	19–65 years	BHW: 42.76±14.11 years SMART: 41.0 ± 14.20 years	To compare the benefits of a strategy-based cognitive training program (SMART) versus an active learning program (Brain Health Workshop- BHW) on cognitive, psychological, and neuroimaging outcomes in adults with traumatic brain injury.

Note:^1^Study’s recruitment age criteria refer to the inclusion criteria specified by the original studies. ^2^Actual participant age is reported separately as mean ± SD or group means, according to the information available. ^3^This study recruited participants aged ≥50 years and was retained as a borderline inclusion because of its direct relevance to the review’s population, concept and main aim.

Abbreviations: Standard Deviation (SD); Metacognitive Training in MS (MaTiMS); Randomised Controlled Trial (RCT); Mild Cognitive Impairment (MCI); Multiple Sclerosis (MS); Huntington’s Disease (HD); Human Immunodeficiency Virus (HIV); China Health and Nutrition Survey (CHNS); Mediterranean healthy eating, ageing, and lifestyle (MEAL); Biomarkers of Cognitive Decline Among Normal Individuals (BIOCARD); Brain Health Workshop (BHW); Strategic Memory Advanced Reasoning Training (SMART).

#### 3.2.1 Cognitive heterogeneity across studies

The aetiology and level of cognitive impairment diagnosis significantly shaped the interpretation and applicability of the findings. Participants spanned a wide range of underlying aetiologies, highlighting that mNCD is not a monolithic condition, but rather a heterogeneous diagnosis. mNCD aetiologies included neurodegeneration, such as MCI [41,43,51,52], secondary to systemic disease, such as Cancer-Related Cognitive Impairment (CRCI) and HIV-associated Neurocognitive Disorder (HAND) [48,55], specific neurodegenerative disorders, such as Multiple Sclerosis (MS) [46,47] and Huntington’s Disease (HD) [49], and brain injury [53,54]. Given this heterogeneity, findings were interpreted within each study’s clinical context and synthesised descriptively rather than treated as a clinically homogeneous group. An example is the vascular and metabolic improvements noted in miners with hypertension [45], which were regarded as highly beneficial for that population, but irrelevant to the metacognitive deficits observed in the amnestic MCI population [51]. Most studies targeted mild impairment, while O’Neil-Pirozzi & Hsu [54] specifically examined individuals with chronic moderate-to-severe brain injury-related cognitive impairment.

### 3.3 Brain health strategies

In this review, ‘brain health strategies’ was the umbrella term used for actions intended to support brain health among middle-aged adults with mNCD, including both formalised interventions (structured programmes delivered or evaluated by researchers), and self-directed practices adopted by individuals. All studies were mapped to modifiable risk factors [9]. Although 14 modifiable risk factors are known, only 4 were directly addressed, and another 3 were addressed as only secondary components. Hearing and visual loss were included only as basic eligibility criteria for participation, with researchers ensuring that participants had adequate sensory function to avoid confounding cognitive testing [39,51]. Air pollution, an emerging risk factor for cognitive decline, as well as LDL cholesterol, traumatic brain injury and low education, were not examined in the studies.

#### 3.3.1 Formalised interventions

Physical inactivity, social isolation, and depression were the most targeted modifiable risk factors in this group. Structured exercise interventions included a 16-week aerobic exercise programme [39], a 12-week dual-task training programme involving dance with simultaneous cognitive tasks [43], and a 12-week moderate-intensity aerobic cycling programme [55]. Across these studies, improvements in cognitive function and related biomarker were reported, with Fitriana et al. [39] reporting significantly lower Apolipoprotein E (ApoE) levels in women with MCI and Nweke et al. [55] reporting a five-fold improvement in working memory of individuals with HAND compared with control groups. Although a limited benefit for working memory was shown in Parial et al.’s [43] study, immediate and delayed memory improved for participants with MCI post-intervention. A longer 2-year multi-component lifestyle programme targeting hypertension incorporated exercise, glucose control, smoking, and alcohol modification, and demonstrated improvements in blood pressure, working memory, and cognitive function over time [45].

Social isolation was addressed as a secondary modifiable risk factor through cognitive training and rehabilitation interventions. These included an RCT comparing computerised cognitive training and music therapy in individuals with HD [49], a virtual-reality cognitive rehabilitation programme with multisensory stimulation for individuals with MS [47], a computerised cognitive programme from BrainHQ [54], and metacognitive or reasoning-based training in individuals with brain injury [53]. These studies reported improvements in selected cognitive or executive outcomes within their respective clinical populations. In addition, increased connectivity between executive-control and sensorimotor networks, as well as abstract reasoning, a skill crucial in daily life for stimulating frontal lobe functions, was reported by Vas [53] and Moreu-Valls et al. [49]. Some interventions inherently involved group settings, providing social interaction as an added benefit, allowing participants to share experiences [43,46,53]. In addition, group-based interventions often reported that psychosocial improvements were linked to social interaction. For example, the dual-task Zumba intervention reported improved quality of life, which could partly stem from the social nature of dancing together [43].

Mindfulness-based interventions targeted depression and psychological well-being. These were added to existing programmes rather than being stand-alone interventions, such as psychoeducation with reflection for individuals with CRCI [48] or the Metacognitive Training in MS (MaTiMS) programme for individuals with MS, which is explicitly aimed at managing neuropsychiatric symptoms [46]. Although objective test scores did not improve, participants reported improved perceived cognitive function, stress, mood, and self-efficacy.

#### 3.3.2 Self-directed practices

Observational studies predominantly captured self-directed practices through naturally occurring dietary patterns, compensatory memory tactics, and lifestyle behaviours. Dietary strategies were examined exclusively through naturally occurring eating habits, including longitudinal analyses of whole-egg consumption [40], aquatic foods and omega-3 fatty acids [44] and adherence to a culturally adapted Mediterranean-DASH Intervention for Neurodegenerative Delay (MIND) diet [42]. Beyond specific nutrients, an Italian study examined the relationship between a self-directed time-restricted eating pattern and cognition [50]. Although these studies reported that healthier dietary patterns and eating behaviours were associated with better cognitive outcomes, they did not support any modifiable risk-reduction claims, and the findings were interpreted as associations rather than effects.

Self-reported engagement in cognitively stimulating leisure activities and the use of compensatory strategies were associated with better outcomes. People living with amnestic MCI reported using compensatory strategies, such as external aids (lists, notebooks, calendars, written notes and reminders) and readily deployable tools to reduce everyday cognitive burden and preserve their independence [51]. Questionnaires were used to examine habitual activity as part of participants’ overall lifestyle engagement [41,52]. Longitudinally, higher engagement in physical, cognitive and social activities was associated with slower cognitive decline among individuals on a dementia trajectory, whereas no association was observed among those who remained cognitively normal [52]. Chen et al. [41] examined a multi-domain self-management profile (healthy diet, exercise, cognitively stimulating activities and social engagement) with caregiver support factors for individuals with MCI. Rather than an outcome, social support was treated as an influencing factor on patients’ self-management approach to adhering to a risk-reduction lifestyle.

### 3.4 Facilitators and barriers identified across strategies

Table 2 outlines the key facilitators and barriers noted by participants and researchers across all studies. Multiple patterns emerged regarding the facilitators and barriers that shaped the success, adherence, and feasibility of strategies. These factors varied according to population characteristics, strategy type, and duration.

**Table 2:** Overview of the modifiable risk factors addressed, strategies, and key barriers and facilitators identified, organised alphabetically.

Authors	Modifiable Risk factors^1^ addressed	Description of strategies^2^	Type of strategy	Duration	Key barrier(s) identified	Key facilitator(s) identified
An & Wang	Did not directly target MRFs	Participant-reported habitual egg consumption frequency	Self-directed: Diet		Self-reported recalls were an imprecise measure of habitual intake due to temporal variations and introduced measurement bias	Longitudinal design enabled exploration of the long-term effects of diet in a large sample.
Baetge et al.	Depression	MaTiMS comprised of modules on memory, attention, depression, fatigue, stress and social cognition, along with psychoeducation and coping strategies. MaTiMS-modified added a neuroeducation module on brain function and 10-minute mindfulness exercises at the end of sessions	Formalised intervention: Psycho-education	MaTiMS: six 90-minute modules over 3 weeks. MaTiMS- modified had seven 100-minute modules over 4 weeks, with follow-up surveys at 3, 6 & 12 months.	COVID-19-related logistical and technical constraints created obstacles to online module access. 15 of 65 patients dropped out due to relapses or treatment.	The intervention was short and flexible, enabling patients to participate with minimal time burden. Many valued the peer engagement, which they felt increased adherence.
Bampa et al.	Did not directly target MRFs	Cognitive compensation strategies and self-efficacy beliefs	Self-directed: Memory aid strategies		Participants were highly educated and small in sample, which may limit the generalisability of the findings to broader populations.	None identified
Chen et al.	Social isolation Physical inactivity	Survey on the multi-domain self-management lifestyle profile	Self-directed: Diet, exercise, social interactions, lifestyle behaviour change		Reliance on self-reported data may introduce potential reporting bias from individuals who have a cognitive impairment.	1. Caregiver modelling resulted in higher caregiver-patient mutuality to adopt brain-healthy lifestyle choices.
Currenti et al.	Did not directly target MRFs	Eating-timing pattern and chrono-nutrition	Self-directed: Diet		Participants were more health-conscious and had higher socio-educational status, which potentially inflates the protective effect attributed to time-restricted feeding	Statistical models used in the study accounted for numerous potential confounding factors, including demographic, lifestyle, and health history variables.
Fitriana et al.	Physical inactivity	Aerobic exercise and dance styles, namely brain gymnastics, poco-poco gymnastics, cardiovascular gymnastics, and rheumatism exercises	Formalised intervention: Aerobic exercise	Twice weekly for 16 weeks (60 minutes), including 10-minute warm-up; 40-minute aerobic core; 10-minute cool-down.	About 20% of participants dropped out due to illness or an inability to attend all 32 sessions.	The program used varied aerobic movement types (multiple routines)
Li et al.^3^	Hypertension; Physical inactivity; Smoking^5^; Diabetes^5^ Alcohol^5^	Lifestyle behaviour-change intervention (including health education, behavioural counselling, health seminars and free educational materials), group sports activities (basketball, badminton, table tennis or mountain climbing) and dietary advice (including salt reduction, weight control, smoking and alcohol cessation)	Formalised multi-component intervention: aerobic exercise, diet & lifestyle behaviour-change	2-year study: 3 monthly health seminars, monthly texts & calls about changing behaviours, with sports activity on Thursday afternoons	Meaningful cognitive benefits emerged only after 1 year of intervention. Some participants were not taking antihypertensive medication due to economic constraints.	Participation supports included the programme’s repeated reinforcement, easy access to educational materials, and the implementation of lifestyle changes in the workplace.
Li et al.^4^	Did not directly target MRFs	Participant-reported food frequency of aquatic food and long-chain omega-3 intake	Self-directed: Diet		Reliance on self-reported data may introduce potential recall bias from individuals who have a cognitive impairment.	None identified
Lu et al.	Did not directly target MRFs	Participant-reported food frequency intake of the MIND diet	Self-directed: Diet		The sample was heavily skewed toward females and drawn from urban square-dancing groups.	Adapting the MIND diet to Chinese dietary habits enhanced its cultural and nutritional relevance.
Maggio et al.	Did not directly target MRFs	Semi-immersive VR training, including cognitive rehabilitation tasks within interactive scenarios and body movement tracking to control the activities and provide real-time audiovisual feedback, while also targeting participants’ attention, visuospatial skills, memory, and executive functions	Formalised intervention: Cognitive training	3 sessions per week, each lasting 60 minutes for 8 weeks (24 sessions)	Eligibility constraints (e.g., exclusion of people with disabling visual/ auditory disturbances or severe disability) acted as real-world barriers.	All participants completed training without side effects, supporting good tolerability.
Melis et al.	Depression; Physical inactivity	Mindfulness program combining guided practices (e.g. breathing meditation, mindful movement, yoga & walking meditation) with psychoeducation, group reflection, and structured daily home practice	Formalised multi-component intervention: Mindfulness and psycho-education	4 group sessions (3 hours each), spread over 8 weeks, and online support	Time burden: 4 participants dropped pre-baseline due to lack of time. Low home-practice adherence to daily mindfulness.	Intentionally reduced number and length of sessions to facilitate participants. Trainers contacted absentees for an update and encouraged them to continue home practice.
Moreu-Valls et al.	Did not directly target MRFs	Computerised cognitive training (NeuronUP®), involving over 250 tasks developed by neuropsychologists Neurologic Music Therapy	Formalised intervention: Cognitive training	Once per week, for 24 weeks (45-minute sessions)	The small sample sizes (around 13–16 per arm) reflect the challenge of enrolling and keeping patients with HD in lengthy interventions	Interventions were highly personalised, e.g., music therapy sessions were customised to each patient’s musical preferences. Deliberate variation of tasks across weeks was explicitly used to maintain engagement.
Nweke et al.	Physical inactivity	Moderate intensity aerobic exercise using a Cycle Ergometer to gradually increase the speed based on participants’ heart rate	Formalised intervention: Aerobic exercise	Moderate intensity aerobic exercise (20–60 minutes), 3 times per week for 12 weeks	Low adherence (under 75% attendance) despite supportive incentives	Support for continued participation was provided, such as incentives, and arranging to train at clinics closer to participants’ homes
O' Neill & Hsu	Did not directly target MRFs	Computerised cognitive exercise programme from BrainHQ, targeting six cognitive categories (e.g., Attention, Brain Speed or Memory)	Formalised intervention: Cognitive training	5 days per week, for 5 months (up to 98 sessions, each lasting 60 minutes)	Participants disliked repetition and timed tasks, perceived some exercises as pointless, and reported fatigue and competing life demands. Adherence was disrupted by health issues such as appointments, illness or hospitalisation.	Participants reported that the program was easier to stick with when they experienced a sense of accomplishment or found the exercises relaxing. The research assistant’s ongoing cueing provided reminders to start and motivation to persist when low interest arose.
Parial et al.	Physical inactivity; Social isolation	Dual-Task Zumba Gold combining low-impact aerobic dance with concurrent cognitive tasks (e.g., orientation, attention, serial counting, recall or spelling), with task difficulty progressing every four weeks.	Formalised multi-component intervention: aerobic exercise & cognitive training	3 times per week for 12 weeks (60 minutes per session)	Some participants faced challenges with attendance and time commitment.	The programme was tailored to older adults’ abilities and interests. Travel allowance was provided.
Pettigrew et al.	Did not directly target MRFs	Participant-reported frequency of lifestyle (physical, cognitive and social) activities	Self-directed: Exercise and social activities		Participants were primarily white, highly educated, and self-selected (strong family history of dementia), limiting generalisability.	Longitudinal follow-up (mean 14.3 years) allowed tracking of preclinical cognitive decline.
Vas et al.	Did not directly target MRFs	SMART programme: metacognitive strategies to improve cognitive control functions of strategic attention, integrative reasoning, and innovation. BHW: new learning about brain health and brain injury	Formalised intervention: Cognitive training	12 group sessions over 8 weeks (18 hours total); post-intervention and 3-month follow-up	Enrolling adults with chronic brain injury required substantial advocacy and recruitment efforts by researchers.	Intensive support for adherence, such as researchers emphasising completing homework. Offering makeup sessions for missed classes.

Abbreviations: Metacognitive Training in MS (MaTiMS); Huntington’s Disease (HD); Brain Health Workshop (BHW); Strategic Memory Advanced Reasoning Training (SMART); Mediterranean-DASH Intervention for Neurodegenerative Delay (MIND).

1 Modifiable risk factors as determined by Livingston et al. [9].

2 Some intervention studies included more than one intervention or compared two interventions.

3 Refers to Li et al. [45].

4 Refers to Li et al. [44].

5 Smoking, alcohol and glucose control were secondary modifiable risk factors addressed as part of the intervention.

#### 3.4.1 Facilitators

A key set of facilitators across the studies concerned the accessibility, enjoyment, and contextual fit of the formalised interventions. Programmes that integrated cognitive stimulation into socially meaningful activities, such as dual-task Zumba Gold [43], semi-immersive virtual reality training [47], and customised music therapy [49], often reported high participant satisfaction, positive mood effects, and high session completion rates. These formats not only stimulated cognitive functions but also enhanced motivation through enjoyment and peer interactions, both of which are critical to strategy adherence and reframing cognitive challenges in less stigmatising terms for individuals with mNCD. Similarly, the delivery flexibility of an online intervention proved especially valuable during the COVID-19 pandemic and for participants with mobility limitations [46]. Melis et al. [48] also proactively reduced the number and length of mindfulness sessions to prevent dropout. In a different context, Nweke et al. [55] improved adherence to aerobic exercise by relocating exercise locations closer to participants' residences, thereby reducing transportation barriers. Researcher facilitation and deliberate adherence management were also critical facilitators in formalised interventions. Facilitation cues and guidance, regular messaging or calls, proactive management of non-attendance (e.g., ‘make-up appointments’ to accommodate those who could not attend), researchers following up on missed sessions, and encouraging participants to complete homework tasks contributed to higher participation and attendance rates [39,46,49,53,54].

Other studies demonstrated that integration into existing support systems can further improve the success of strategies. Caregiver involvement, defined as participants whose caregivers participated in brain-healthy behaviours, was associated with modelling and improved adherence to brain-healthy lifestyle changes among individuals with MCI [41]. Similarly, lifestyle changes implemented in a workplace setting over multiple years facilitated participation by integrating formalised interventions into a routine occupational health system, thereby reducing reliance on self-initiation alone [45]. At a broader psychological level, stronger individual health beliefs, preserved awareness, self-control, and motivation to cope with cognitive difficulties facilitated the adoption and maintenance of lifestyle or cognitive strategies. Individuals with amnestic MCI reported using memory recall strategies at rates similar to those of healthy controls [51], suggesting that, despite objective impairments, people with MCI may retain a high level of strategic engagement, particularly when strategies are familiar or socially normalised. In addition, those who believed they could still reduce their risk of dementia were more likely to use compensatory strategies such as enhanced self-monitoring, grocery planning, or dietary consistency [42].

#### 3.4.2 Barriers

The most prominent barriers included health-related limitations, time burden, logistical constraints, and underlying socioeconomic factors. In studies involving populations with chronic conditions, health-related obstacles presented recurrent issues in formalised interventions, with a quarter of participants withdrawing due to disease relapse or treatment [46] and missing sessions due to scheduling conflicts with medical appointments and intercurrent illness [54]. Similarly, health issues and competing role demands saw nearly half of the eligible women with CRCI decline enrolment or show low adherence to integrating strategies into daily home life [48]. In addition, high cognitive load and increased session duration were identified as barriers to participation in intervention studies. Participants reported that their concentration waned as sessions progressed and recommended shorter sessions with more frequent breaks to prevent fatigue [43,46,48]. Despite designing interventions to leverage procedural learning by implementing a fixed routine, participants did not initiate sessions independently and required additional cueing or supervision [43,53,54]. These studies noted that relying on self-initiation, even with a consistent schedule, was unrealistic for many cognitively impaired individuals unless strong external prompting structures were in place. Conversely, another study involving participants with HD reported high adherence and satisfaction with all activities, despite randomisation that led to one intervention arm beginning with participants with more severe cognitive impairment [49].

In Baetge et al.’s [46] study, transitioning the intervention online due to COVID-related logistics created barriers, including limited internet access, device availability, and reduced personal connection in group sessions. Similarly, in Nweke et al.’s [55] study, long travel distances to exercise centres hindered participation, prompting referrals to clinics closer to participants’ homes, however, adherence remained below 75% despite these efforts. At the socioeconomic and belief level, lower income and low social support were associated with poorer adoption of healthy lifestyles in individuals with MCI, indicating real-world resource constraints within this population [41,51]. Barriers to self-reported practices were frequently rooted in low self-efficacy and limited understanding of effective techniques. Participants with MCI who used memory-aid strategies rated themselves as significantly less confident in their ability to apply those strategies effectively in real-world settings [51]. Some participants also reported embarrassment and social discomfort when struggling with cognitive tasks in group settings, which led to dropout and avoidance of self-guided practice [43]. Furthermore, even promising lifestyle changes encountered behavioural resistance. Most participants did not naturally follow an early-time-restricted eating window, suggesting that dietary timing changes may be difficult to implement without targeted behavioural support [50]. Similarly, Li et al. [44] acknowledged that, despite positive cognitive associations, participants’ concerns about mercury exposure from fish consumption could deter long-term dietary adherence.

### 3.5 Equity across studies

Across included studies, equity-relevant characteristics mapped to PROGRESS-PLUS were most often reported descriptively and less often used analytically. The ‘Plus’ factors were integral to every study because samples were defined by cognitive impairment, and this scoping review examined a specific age range. However, most PROGRESS characteristics were inconsistently measured and rarely used to test differential effects or guide analysis. A clear pattern emerged by study type, with larger studies examining self-directed practices having more comprehensive equity coverage and adjusting for core social variables (typically age, sex, and education) [40,42,50], and one study explicitly incorporating social support and caregiver characteristics into brain health lifestyle modelling [41]. In contrast, many formalised intervention studies treated equity characteristics primarily as descriptive background variables or eligibility criteria, such as excluding individuals with sensory impairment or severe depression, rather than as explanatory variables [39,43,51,52]. Table 3 outlines the PROGRESS-Plus characteristics of all studies.

**Table 3:** PROGRESS-Plus characteristics across all studies, ranked according to the most equity coverage.

Author(s); Country	Place of residence	Race/ ethnicity/ culture/ language	Occupation	Gender/ sex	Religion	Education	Socio-economic status (SES)	Social capital (social relations)	PLUS Factors
Chen et al., China	Participants recruited in China; Majority of participants lived in urban areas (>80%) vs rural areas (<17%)	Chinese adults; questionnaires translated into Mandarin	Patients unemployed 90.1%, employed 9.9%; Caregivers unemployed 69.5%, employed 30.5%	Patients: 29.1% male, 70.9% female; Caregivers: 59.6% male, 40.4% female	Not reported	Patients & caregivers categorised as junior high or lower, high/ vocational, or university level	Monthly individual income in RMB yuan categorised as 0–4999, 5000–9999 & >10,000	Perceived Social Support Scale used to measure social relation	Age, relationship & chronic disease status included
Vas et al., USA	Dallas, Texas, USA	Native English speakers; ethnicity not reported	Employment impact noted: Many had difficulty returning to work or getting jobs	53% male; 47% female	Not reported	Mean of 17 years in both groups	SES mean index of 48–50%	Support groups for veterans	Age & head injury as disability status included
Currenti et al., Italy	Southern Italy (Catania districts)	Italian adults; ethnicity not specified	Categorised as unemployed, low, medium, and high	501 female; 382 male	Not reported	Collected as low, medium and high education levels	Collected as un-employed, low, medium & high occupation status	Not reported	Age & chronic disease status included
Lu et al., China	Residents in urban areas in Wuhan, Yichang, Xiangyang, Shanghai, Xiamen, Beijing, Chengdu	Chinese adults; language not specified	Not reported	85.07% female, 14.9% male	Not reported	12.83% below primary, 75.46% middle school, 11.72% high school or above	Income levels collected: <20,000 CNY^4^ 20,000–39,999 CNY; ≥40,000 CNY	Participants attended Dance clubs	Age & chronic disease status included
Nweke et al., Nigeria	Nigeria (University of Nigeria Teaching Hospital cohort)	Nigerian adults; language & ethnicity not explicitly discussed	Categorised as: farming, public servant, trading, cleaning, self-employed, unemployed & retired.	Majority female; 28–29 females compared to 7–9 males; across both groups	Not reported	Categorised as primary, completed secondary (most participants) and incomplete secondary	Not reported	Married status in intervention group: 68% & control group: 43%	Age & HIV health status included
An & Wang, China	15 provinces/ municipal cities with diverse urban & rural geography	Chinese older adults; language not specified	Not reported	49% male	Not reported	27% had primary school or above	health insurance coverage used as SES indicator (40% insured)	Not reported	Age & chronic disease included
Baetge et al., Germany	Germany	German-speaking participants; ethnicity not reported	18 participants were in employment, but no details are given	77% & 92% female across both intervention arms	Not reported	58–62% reported "high" education	Not reported	Not reported	Age & Multiple Sclerosis as disability status included
Melis et al., Belgium	Leuven, Belgium	Native Dutch speakers; ethnicity not specified	recorded about 74–75% employed across groups	100% female	Not reported	Secondary school: Mindfulness group: 28% Physical training group: 31% Higher education: Mindfulness group: 72% Physical training group: 69%	Not reported	Not reported	Age & Cancer health status included
Li et al., China^1^	Community-dwelling adults in Wuhan, Yichang, Xiangyang, Shanghai, Xiamen, Beijing, Chengdu	Chinese adults; language not specified	Not reported	85.73% female	Not reported	8- 17% had 0–6 years; 75–80% had 7–12 years; 7–14% had >12 years education	Not reported	Marital status “with partner” or “without a partner” recorded	Age & chronic disease included
Li et al., China^2^	Shaanxi province Jinduicheng and Hancheng mining areas.	Chinese adults; language not specified	Mine workers	74.9% male; 25.1% female	Not reported	Mean 10.6 ± 1.8 years	Not reported	Not reported	Age & chronic disease included
Fitriana et al., Indonesia	2 urban community settings in Indonesia	Indonesian language; ethnicity not specified	Not reported	100% female	Not reported	Recorded as low (elementary/ junior high) vs high (senior high/ university)	Not reported	Marital status collected, but not reported	Age included
Bampa et al., Greece	Day Care centre in Greece	Native Greek speakers; ethnicity not reported	Not reported	33% male, 67% female	Not reported	Mean 14.95 years across groups	Not reported	Not reported	Age included
Maggio et al., Italy	One rehab centre in Messina, Italy	Italian language; ethnicity not specified	Not reported	51.7% male, 48.3% female	Not reported	37% middle school, 53% high school; 10% university	Not reported	Not reported	Age & Multiple Sclerosis health status included
Moreu-Valls et al., Spain	Single centre in Barcelona, Spain	Spanish language; ethnicity not reported	Not reported	22 females (roughly balanced with males)	Not reported	high mean of 12–15 years across all groups	Not reported	Not reported	Age & Huntington’s Disease as a disability status included
O'Neil-Pirozzi & Hsu, USA	MA, USA	Not discussed	Not discussed	79% male; 29% female	Not reported	10 high school graduates & 4 had some college	Not reported	living in an assisted living facility- existing social structure that can provide day-to-day support	Age & brain injury as a disability status included
Parial et al., Philippines	Bulacan province, Philippines	Not reported	Not reported	76.7% female; 23.3% male	Not reported	28.3% elementary, 53.3% secondary, 18.3% college	Not reported	70% married, 3.3% single, 6.7% separated/ divorced, 20% widowed	Age & health status included
Pettigrew et al., USA	USA (John Hopkins University cohort)	97.4% White; English language	Not reported	60.8% female	Not reported	Highly educated (mean 17.4 years)	Not reported	Not reported	Age, genetic family history & chronic disease status included

1 Refers to Li et al. [44].

2 Refers to Li et al. [45].

By PROGRESS characteristics, place of residence was predominantly urban, either in clinical centres, health-engaged community settings, or assisted living facilities, and only one study was based in a rural industrial setting, explicitly anchoring risk and intervention feasibility within workplace-based conditions [45]. Equity patterns varied systematically across countries or health systems, although race and ethnicity were rarely characterised beyond the country or language-level. Cultural specificity was largely inferred and adapted through local adaptation, such as the Chinese-adapted MIND diet, which accounts for the traditional consumption of freshwater fish [42], in contrast to Western staples such as wine, dairy, and olive oil [50]. Cognitive impairment commonly intersected with systemic or occupational risks, such as HAND in Nigerian adults across diverse low- and middle-status occupations [55], or hypertension in Chinese mining communities [45], compared to High-Income Countries (HICs), where it was more often framed as a function of ageing [51], lifestyle [50] or treatment effects [48]. In East Asian and Low- and Middle-Income countries (LMICs), strategies were often framed around familial responsibility and community-based peer support. An example is the dyadic design that explicitly models caregiver characteristics and perceived social support as key determinants of a patient’s lifestyle [41].

Gender representation was frequently biased by recruitment context, with samples being heavily female-dominated, particularly from dance studies or those having breast cancer [42,44,48], and male predominance in specific mining occupational cohorts [45]. Only a few studies examined gendered differences in adherence or outcomes. Education was commonly captured but seldom used beyond adjustment despite its centrality to cognitive reserve and intervention feasibility. Many cohorts in the HICs were highly educated to university level [48,52,53], while some cohorts from East Asian communities also had substantial secondary or higher education [41,44], raising concerns about external validity for less educated populations. Similar to education, socioeconomic status was measured through income, insurance coverage, or occupation and reported in only four studies [40–42,50], limiting the interpretation of equity in access, adherence, and benefit from strategies. Lower educational attainment was strongly linked to lower cognitive scores, less healthy lifestyles, and reduced motivation to engage in brain health behaviours in Chinese cohorts [40]. Conversely, higher income levels or health insurance coverage correlated with better cognitive test scores and a greater inclination to adopt dementia risk-reduction lifestyles [41]. Finally, social capital was most often reported as a secondary component, either through perceived social support scales [41], lifestyle questionnaires that included social engagement [52], or through incidental social contact in formalised group-based interventions [39,43,46,53]. The ‘Plus’ factors of disability and disease status were typically defined by single aetiologies (MS, HD, CRCI, HAND, TBI or MCI), which were appropriate for condition-specific efficacy testing but limited cross-condition comparisons.

## 4 Discussion

The strategies used by middle-aged adults with mNCD to maintain brain health predominantly involved risk-factor modification, cognitive reserve enhancement, and compensatory supports for everyday functioning. This clustering is broadly consistent with contemporary framings of brain health as multidomain functioning shaped by continuous interaction between biology and environment across the life course [7]. Physical inactivity was the most commonly represented modifiable risk factor. Several formalised intervention studies reported improvements in selected cognitive outcomes, while observational studies identified associations between habitual activity and cognitive trajectories [39,43,52,55]. However, consistent with other studies [56,57], effects were domain-specific and sometimes delayed, highlighting the need for longitudinal measurements of physical activity and clearer activity-response optimisation for middle-aged individuals. These findings, therefore, should not be interpreted as evidence of effectiveness because the included studies differed substantially in design, population, intervention content and outcome measurement. Cardiometabolic prevention research was also identified [45,50] but remained scarce relative to its epidemiological importance. This scarcity is difficult to justify given that midlife cardiovascular risk factors, particularly hypertension and metabolic control, are repeatedly described as major factors in cognitive trajectories and dementia risk [30,[58], [59], [60]]. It also represents a missed opportunity given the ‘equity promise’ of scalable, primary-care-based vascular risk management [61]. The epidemiological salience of midlife vascular risk and its comparatively limited representation in this review suggests a translational gap rather than a knowledge gap. Diet strategies were methodologically bifurcated in observational studies [40,42,44], which aligned conceptually with cardiometabolic-related neurodegenerative risk, yet, similar to other studies [62,63], they remain better interpreted as a cognition risk association rather than a risk-reduction claim. This highlights a recurring interpretive slippage in brain health messaging and research translation, in which observational associations are sometimes presented as causal evidence for prevention.

Consistent with other research [64,65], some studies reported more favourable outcomes among individuals identified as high-risk or already experiencing cognitive decline, suggesting a cognitive reserve mechanism in those with this pathology [52]. However, this conflicts with the composition of many study participants, who were relatively able to engage with structured programmes, thereby inflating perceived feasibility and obscuring structural constraints. Thus, the populations for whom strategies may be most clinically relevant are not necessarily the ones most represented.

Equity considerations further clarify why feasibility and effectiveness cannot be separated. PROGRESS-PLUS characteristics were often identified descriptively and did not explicitly inform studies or strategies, contrary to the recommendation of applying an equity lens when designing and evaluating strategies [66]. Strategies reliant on literacy, technology, or complex multi-component approaches [46,47,54] risked exacerbating inequities and inequalities among individuals with lower socioeconomic status, lower education, and those with complex diseases, precisely the populations most likely to face constrained opportunities for sustained behavioural change [61,67]. In addition, formalised interventions targeted modifiable risk factors, whereas the most powerful facilitators and real-world constraints remained at the social and structural levels of the midlife life course.

Intensive multi-week training, structured exercise sessions, lengthy programmes and travel requirements were frequently indicated with adherence difficulties, including dropout, non-attendance and scheduling conflicts. In this context, these are often better interpreted as an intervention–context mismatch rather than as individual-level adherence failure, indicating that the participation burden itself functions as an equity mechanism. This is reinforced by Migeot et al. [68], who argue that, under adverse social conditions, even non-monetary costs, such as time and effort, become under-recognised and unequally distributed social determinants of brain health. Several studies often implicitly assumed time commitments from participants in their formalised interventions [47,49], yet temporal inequity, defined as unequal access to and control over discretionary time, functioned as a social determinant that constrained participation and engagement [69]. This can be interpreted as high-agency strategies may be least accessible to those most structurally exposed to dementia risk, making feasibility a core equity outcome rather than a secondary implementation detail.

Similarly, Daly’s conceptualisation of the ‘iceberg of dementia risk’ emphasises that addressing only lifestyle behaviours and strategies is inadequate, given the impact of structural determinants and deprivation from economy, housing, work, and education [70]. This was evident in Li et al.’s [45] study, which showed participants receiving lifestyle advice and interventions, but some were unable to afford their antihypertensive medications, thereby leaving a primary modifiable risk factor unaddressed. Public health messaging intended to promote brain health has also overwhelmingly centred on high-agency individual strategies and has inadvertently reinforced culpability for communities whose risk distribution reflects unresolved structural inequalities rather than individual fault [71]. Furthermore, a systematic review on brain health found that most samples were drawn from relatively advantaged, WEIRD (Western, Educated, Industrialised, Rich, Democratic) settings and trial cohorts [72], echoing broader concerns that populations from minority communities and LMICs are underrepresented in both observational and interventional studies [5,14]. Consistent with this, studies included in this scoping review found participants were mostly educated and predominantly from urban areas in seven HICs. Finally, it is notable that no included study explicitly reported participant co-design; instead, all were based on existing evidence or researcher-designed programmes.

Overall, the predominance of condition-specific samples, the frequent reliance on urban, educated, and physically active participants, and the absence of core social determinants limit the ability to identify which subgroups may experience more favourable outcomes, which remain underserved, and how to tailor multidomain brain health strategies. In particular, there is a dearth of studies exploring the lived experiences of middle-aged individuals with mNCD, and even fewer adopt an explicit equity lens despite growing recognition that equity factors shape opportunities to engage in brain health behaviours.

### 4.1 Implications for future works

Future research should shift from isolated efficacy studies to pragmatic, co-designed, implementation-focused works that test how brain health strategies can be embedded within schools, workplaces, primary care, and community services. It should also move beyond descriptive reporting towards intentional, equity-informed sampling and analysis, and should evaluate and tailor strategies for effectiveness, feasibility, and equity across clinically and aetiologically heterogeneous populations and settings. More importantly, future brain health research needs to extend beyond individual-level lifestyle framings to evaluate structural interventions that explicitly address deprivation, nonmonetary costs, and broader social determinants. There is also a need for studies to better capture the contextual variability of ‘midlife’. Rather than relying on a single age interval, studies should justify their operational definition and report sufficiently granular age information to allow re-aggregation across alternative definitions in subsequent evidence syntheses.

In practical terms, future work can involve tailoring programmes for those of lower socioeconomic status, adapting materials for different literacy levels, offering low-tech and culturally adapted options, and partnering with community organisations that have trusted relationships with underserved populations and those with existing mNCD to co-create solutions. Finally, at policy level, evaluation frameworks should mandate reporting of reach, adherence, and outcomes by socioeconomic and demographic indicators to ensure that benefits are distributed fairly across midlife populations.

### 4.2 Limitations

This scoping review provides essential insights into an understudied population, but it has limitations. Although grey literature sources were searched, no eligible grey literature records were identified; therefore, the final evidence base comprised peer-reviewed journal articles. It is thus possible that relevant research available as preprints, conference proceedings, or dissertations may have been missed. Second, this scoping review included studies published from 2015. The search was limited to ensure that the evidence reflects contemporary definitions, methodologies, and technological advances in brain health research, as the past 10 years have marked a shift toward multi-domain prevention strategies and alignment with global policy initiatives. The heterogeneity in the age reporting of the included studies should be considered when interpreting the findings of this review. We operationalised midlife by chronological age, defined as 40 to 65 years; however, studies did not apply uniform age eligibility criteria, and several recruited participants across broader adult age ranges and did not report findings separately for participants within the midlife period. One such study, which reported a mean age of 67.67 years, was retained because its recruitment criteria included participants within the prespecified midlife age range. Nevertheless, the absence of age-disaggregated findings in this study means that the proportion of middle-aged participants could not be established, limiting the extent to which the results can be attributed specifically to midlife adults. Evidence limitations also constrain how assertively recommendations can be made in clinical practice, such as for specific dietary patterns or social connectivity, for which there is insufficient data to justify major changes in guidelines, thereby making clinical messaging more cautious and less directive.

## 5 Conclusion

International programmes and frameworks advocate for brain health through prevention, early intervention, and risk reduction. This scoping review identified a diverse range of formalised interventions and self-directed strategies used by individuals with mNCD across aetiologically heterogeneous populations within the midlife age range. The findings indicate that engagement with these strategies was influenced by a complex interplay of psychological, cognitive, and logistical factors, as well as social determinants of health, while equity considerations were rarely systematically integrated into their design and implementation. The included studies reported outcomes relating to cognitive functioning, psychological well-being, and health-related behaviours, although the nature and extent of reported outcomes varied across population characteristics, intervention designs, and study methodologies. Formalised interventions were implemented primarily in a top-down manner, which may have contributed to recurring barriers related to adherence, accessibility and feasibility. Collectively, these findings provide an important foundation for understanding the breadth and characteristics of current evidence and for identifying strategies that warrant further rigorous and aetiology-specific evaluation during midlife. Future brain health strategies should also prioritise feasibility, cultural responsiveness, equity, and co-production with participants to reflect real-world needs and constraints.

## Funding

Three authors (JD, JH & YMcC) are currently recipients of the SNMHS PhD scholarship from their affiliated university (University College Dublin). The funders had no role in the study design, data collection and analysis, the decision to publish, or the preparation of the manuscript.

## Declaration of generative AI and AI-assisted technologies in the writing process

None

## Ethical statement

Ethical approval is not required.

## Data statement

The data supporting the findings of this study are available within the article and its Supplementary Materials. The full extracted dataset is available from the corresponding author upon reasonable request.

## CRediT authorship contribution statement

**Joshi Dookhy:** Writing – review & editing, Writing – original draft, Project administration, Methodology, Formal analysis, Data curation, Conceptualization. **Yvonne McCague:** Writing – review & editing, Methodology, Formal analysis. **Jim Hickson:** Writing – review & editing, Methodology, Formal analysis. **Diarmuid Stokes:** Software, Resources, Methodology. **Thilo Kroll:** Writing – review & editing, Supervision, Methodology, Conceptualization. **Kate Frazer:** Writing – review & editing, Supervision, Methodology, Conceptualization.

## Declaration of competing interest

The authors declare that they have no known competing financial interests or personal relationships that could have appeared to influence the work reported in this paper.

## Appendix Supplementary materials

Image, application 1
