# Supplementary material for: Brain health strategies in middle-aged individuals with mild neurocognitive disorder: a scoping review
Source: J Prev Alzheimers Dis. 2026 Aug 13;13(9):100657. doi: 10.1016/j.tjpad.2026.100657 (PMC13499126; doi:10.1016/j.tjpad.2026.100657)
Supplement: Supplementary file 1 [file mmc1.docx]

**Supplementary Material**

**Table S1. Search strategy of each database**

| **PubMed (SEARCHED 14/8/25)** | | |
| --- | --- | --- |
| **Search** | **Query** | **Records retrieved** |
| #1  Population | "Cognition Disorders"[Mesh] OR "Amnesia"[Mesh] OR "Delirium"[Mesh]  OR "Cognitive Impairment"[Title/Abstract] OR "Mild Neurocognitive Disorder"[Title/Abstract] OR "MCI"[Title/Abstract] OR "Memory Deficit*"[Title/Abstract] OR "neurocognitive dysfunction*"[Title/Abstract] OR “Cognit* Disorder*"[Title/Abstract] OR "Amnesia"[Title/Abstract] OR "Delirium"[Title/Abstract] | **245,045** |
| #2  Concept | "Cognitive Health"[Title/Abstract] OR "cognitive preservation*"[Title/Abstract] OR "cognitive enhancement*"[Title/Abstract] OR "brain reserve*"[Title/Abstract] OR "brain health"[Title/Abstract] OR "dementia risk* reduction*"[Title/Abstract] OR "Preventive Health"[Title/Abstract] OR "neuroprotecti*"[Title/Abstract] OR "Healthy Aging"[Title/Abstract] OR "Health Status"[Title/Abstract]  OR “Cognitive Reserve"[Title/Abstract] OR  "Cognitive Reserve"[Mesh] OR "Preventive Health Services"[Mesh] OR "neuroprotection"[Mesh] OR "Healthy Aging"[Mesh] OR "Health Status"[Mesh] | **1,382,116** |
| #3  Concept | "health strategy"[Title/Abstract] OR "Cognitive Dissonance"[Mesh] OR "Cognitive Flexibility"[Mesh] OR "Health Promotion"[Mesh] OR "Metacognition"[Mesh] OR "resilience, psychological"[Mesh] OR "Human Activities"[Mesh] OR "Psychotherapy"[Mesh] OR "Health Services"[Mesh] OR "Psychological Phenomena"[Mesh] OR "Life Style"[Mesh] OR "Cognitive Dissonance"[Title/Abstract] OR "Cognitive Flexibility"[Title/Abstract] OR "Health Promotion"[Title/Abstract] OR "Metacognition"[Title/Abstract] OR "resilience"[Title/Abstract] OR  "Human Activities"[Title/Abstract] OR "Psychotherapy"[Title/Abstract] OR "Health Services"[Title/Abstract] OR "Psychological Phenomena"[Mesh] OR "Life Style"[Title/Abstract] OR "health aware*"[Title/Abstract] OR "health improvement*"[Title/Abstract] OR "health intervention"[Title/Abstract] OR "health-related intervention"[Title/Abstract] OR "preventive intervention"[Title/Abstract] OR "public health intervention"[Title/Abstract] OR "health promotion"[Title/Abstract] OR "behavi*ral intervention"[Title/Abstract] OR "lifestyle intervention"[Title/Abstract] OR "wellness program*"[Title/Abstract] OR "disease prevention"[Title/Abstract] OR "clinical intervention"[Title/Abstract] OR "modifiable risks factors"[Text Word] OR "brain health behavi*rs"[All Fields] | **5,347,022** |
| #4 Concept | #2 AND #3 | **960,971** |
| #5 | #1 AND #4 [** No filters or exclusion criteria applied] | **17,761** |
| #6 | #1 AND #4 [**Filters applied from 2015-2025] | **10,195** |
| #7 | #1 AND #4 [**Filters applied: from 2015-2025, English language] | **9880** |
| #8 | #1 AND #4 [**Filters applied: from 2015-2025, English language, Abstract available] | **9630** |
| #9 | #1 AND #4 [**Filters applied: from 2015-2025, English language, Abstract available, Human] | **8196** |
| #10 | #1 AND #4 [**Filters applied: from 2015-2025, English language, Abstract available, Human] *Pubmed specific Articles filters included: Clinical Study, Clinical Trial, Comparative Study, Evaluation Study, Multicenter Study, Observational Study, Randomized Controlled Trial | **1807** |
| **Embase (SEARCHED 14/8/25) *PubMed to Embase search conversion done by mapping the MeSH headings to their EMTREE equivalents** | | |
| **Search** | **Query** | **Records retrieved** |
| #1  Population | 'cognition disorder'/exp OR 'amnesia'/exp OR 'delirium'/exp OR 'cognitive impairment':ti,ab OR 'mild neurocognitive disorder':ti,ab OR mci:ti,ab OR 'memory deficit*':ti,ab OR 'neurocognitive dysfunction*':ti,ab OR 'cognit* disorder*':ti,ab OR amnesia:ti,ab OR delirium:ti,ab | 853,792 |
| #2  Concept | 'cognitive health':ti,ab OR 'cognitive preservation*':ti,ab OR 'cognitive enhancement*':ti,ab OR 'brain reserve*':ti,ab OR 'brain health':ti,ab OR 'dementia risk* reduction*':ti,ab OR 'preventive health':ti,ab OR neuroprotecti*:ti,ab OR 'healthy aging':ti,ab OR 'health status':ti,ab OR 'cognitive reserve':ti,ab OR 'cognitive reserve'/exp OR 'preventive health service'/exp OR 'neuroprotection'/exp OR 'healthy aging'/exp OR 'health status'/exp | 663,094 |
| #3 Concept | 'health strategy':ti,ab OR 'cognitive dissonance'/exp OR 'cognitive flexibility'/exp OR 'health promotion'/exp OR 'metacognition'/exp OR 'psychological resilience'/exp OR 'human activity'/exp OR 'psychotherapy'/exp OR 'health service'/exp OR 'psychological phenomenon' OR 'lifestyle'/exp OR 'cognitive dissonance':ti,ab OR 'cognitive flexibility':ti,ab OR 'health promotion':ti,ab OR 'metacognition':ti,ab OR resilience:ti,ab OR 'human activity':ti,ab OR psychotherapy:ti,ab OR 'health service':ti,ab OR 'psychological phenomenon':ti,ab OR lifestyle:ti,ab OR 'health aware*':ti,ab OR 'health improvement*':ti,ab OR 'health intervention':ti,ab OR 'health-related intervention':ti,ab OR 'preventive intervention':ti,ab OR 'public health intervention':ti,ab OR 'behavior* intervention':ti,ab OR 'lifestyle intervention':ti,ab OR 'wellness program*':ti,ab OR 'disease prevention':ti,ab OR 'clinical intervention':ti,ab OR 'modifiable risk factors':ti,ab OR 'brain health behavior*':ti,ab | 11,132,883 |
| #4 Concept | #2 AND #3 | 342,946 |
| #5 | #1 AND #4 [** No filters or exclusion criteria applied] | **38,468** |
| #6 | #1 AND #4 AND [2015-2025]/py | **27,391** |
| #7 | #1 AND #4 AND [2015-2025]/py AND [english]/lim | **27,067** |
| #8 | #1 AND #4 AND [2015-2025]/py AND [english]/lim AND [abstracts]/lim | **25,517** |
| #9 | #8 AND 'human'/de | **20,051** |
| #10 | #9 AND 'article'/it | **9,654** |
|  | *EMBASE specific Articles filters included (similar to Pubmed): Clinical Study, Clinical Trial, Comparative Study, Evaluation Study, Multicenter Study, Observational Study, Randomized Controlled Trial | **3997** |
| **CINAHL (SEARCHED 14/8/2025)** | | |
| **Search** | **Query** | **Records retrieved** |
| Population | S5 S1 OR S2 OR S3 OR S4  S4 TI neurocogniti* dysfunction* OR AB neurocogniti* dysfunction*  S3 TI mild neurocogniti* disorder* OR AB mild neurocogniti* disorder*  S2 TI cogniti* impair* OR AB cogniti* impair*  S1 (MH "Cognition Disorders") OR (MH "Delirium, Dementia, Amnestic, Cognitive Disorders") OR (MH "Cognition") | **131,590** |
| Concept | S12 S6 OR S7 OR S8 OR S9  S9 TI dementia risk* reduction* OR AB dementia risk* reduction*  S8 TI brain health OR AB brain health  S7 (MH "Healthy Aging") OR (MH "Middle Age") OR (MH "Healthy Life Expectancy") OR (MH "Cognitive Aging")  S6 (MH "Cognitive Flexibility") OR (MH "Cognitive Aging") OR (MH "Cognitive Therapy") | 1,229,291 |
| Concept | S20 S10 OR S11 OR S13 OR S14 OR S15 OR S16 OR S17 OR S18 OR S19  S19 TI cognitive reserve OR AB cognitive reserve  S18 TI lifestyle intervention* OR AB lifestyle intervention*  S17 TI modifiable risk* factor* OR AB modifiable risk* factor*  S16 TI preventive intervention* OR AB preventive intervention*  S15 TI behavi*ral intervention* OR AB behavi*ral intervention*  S14 TI health improvement* OR AB health improvement*  S13 TI disease prevention OR AB disease prevention  S11 (MH "Population Health") OR (MH "Population Health Management") OR (MH "Nursing Interventions") OR (MH "Psychosocial Intervention")  S10 (MH "Health Promotion") OR (MH "Psychological Comfort Promotion (Iowa NIC)") | 179,816 |
| Concept (AND) | S21 S12 AND S20 | 32,851 |
|  | S22 S5 AND S21 [** No filters or exclusion criteria applied] | **1464** |
|  | S23 S5 AND S21 [2015-2025] | **1040** |
|  | S24 S5 AND S21 [2015-2025, English] | **1022** |
|  | S25 S5 AND S21 [2015-2025, English, Abstract] | **972** |
|  | S26 S5 AND S21 [2015-2025, English, Abstract, Article- academic journal]  *All articles were related to only human studies- ‘human’ limiter not applicable.  **EBSCOhost did not detail article subtype | **963** |
| **APA PsycInfo (SEARCHED 14/8/25) *PubMed to PsycInfo search conversion done by mapping the MeSH headings to their EBSCOhost equivalents** | | |
| **Search** | **Query** | **Records retrieved** |
| #1  Population | S3 S1 OR S2  S2 (TI “Cognitive Impairment” OR AB “Cognitive Impairment” OR TI “Mild Neurocognitive Disorder” OR AB “Mild Neurocognitive Disorder” OR TI MCI OR AB MCI OR TI “Memory Deficit*” OR AB “Memory Deficit*” OR TI “neurocognitive dysfunction*” OR AB “neurocognitive dysfunction*”)    S1 DE "Mild Cognitive Impairment" OR DE "Neurocognitive Disorders" OR DE "Cognitive Impairment" | 83,417 |
| #2  Concept | S6 S4 OR S5  S5  DE "Cognitive Reserve" OR DE "Preventive Health Services" OR DE "Neuroprotection" OR DE "Healthy Aging" OR DE "Health Status"    S4  (TI "Cognitive Health" OR AB "Cognitive Health" OR TI "Cognitive Preservation" OR AB "Cognitive Preservation" OR TI cognitive enhancement* OR AB cognitive enhancement* OR TI "Brain Reserve" OR AB "Brain Reserve" OR TI "brain health" OR AB "brain health" OR TI "dementia risk reduction" OR AB "dementia risk reduction") | **43,274** |
| #3  Concept | S9 S7 OR S8  S7  (TI("health strategy") OR AB("health strategy")) OR (TI("health aware*") OR AB("health aware*")) OR (TI("health improvement*") OR AB("health improvement*")) OR (TI("health intervention") OR AB("health intervention")) OR (TI("health-related intervention") OR AB("health-related intervention")) OR (TI("preventive intervention") OR AB("preventive intervention")) OR (TI("public health intervention") OR AB("public health intervention")) OR (TI("health promotion") OR AB("health promotion")) OR (TI("behavioral intervention") OR AB("behavioral intervention")) OR (TI("lifestyle intervention") OR AB("lifestyle intervention")) OR (TI("wellness program") OR AB("wellness program")) OR (TI("disease prevention") OR AB("disease prevention")) OR (TI("clinical intervention") OR AB("clinical intervention")) OR (TI("modifiable risk factor*") OR AB("modifiable risk factor*")) OR (TI("brain health behaviors") OR AB("brain health behaviors"))  S8  DE "Cognitive Dissonance" OR DE "Cognitive Flexibility" OR DE "Health Promotion" OR DE "Metacognition" OR DE "Psychological Resilience" OR DE "Human Activities" OR DE "Psychotherapy" OR DE "Health Services" OR DE "Psychological Phenomena" OR DE "Lifestyle" | **176,016** |
| #4 Concept | S10 S9 AND S6 | **4095** |
| #5 | S1 AND S10 [** No filters or exclusion criteria applied] | **183** |
| #6 | S1 AND S10 [Filters applied: 2015-2025] | **125** |
| #7 | S1 AND S10 [Filters applied: 2015-2025, English] | **125** |
| #8 | S1 AND S10 [Filters applied: 2015-2025, English, Human] | **111** |
| #9 | S1 AND S10 [Filters applied: 2015-2025, English, Human, Article- academic journal] *Abstract available as a limiter not available on database  **EBSCOhost did not detail article subtype | **97** |

**Table S2. Articles uploaded onto EndNote 21, prior transfer to Covidence**

| **DATABASE** | **Search results** | **De-duplication** | **Retracted from journal** | **Total Included** |
| --- | --- | --- | --- | --- |
| **PUBMED** | 1807 | 0 | 2 | 1805 |
| **EMBASE** | 3997 | 11 | 2 | 3984 |
| **CINAHL** | 963 | 6 | 1 | 956 |
| **PsychINFO** | 97 | 0 | 0 | 97 |
| **Total** | 6864 | 17 | 5 | 6842 |
